# Effectiveness of Postoperative Physiotherapy Compared to Postoperative Instructions by Treating Specialist Only in Patients With an Ankle Fracture: A Systematic Review

**DOI:** 10.1177/24730114231173680

**Published:** 2023-05-13

**Authors:** Robyn Van Vehmendahl, Stijn D. Nelen, Mouhcine El Hankouri, Michael J. R. Edwards, Albert F. Pull ter Gunne, Diederik P. J. Smeeing

**Affiliations:** 1 Department of Trauma Surgery, Radboud University Medical Centre, Nijmegen, the Netherlands; 2 Department of Trauma Surgery, Rijnstate Hospital, Arnhem, the Netherlands

**Keywords:** ankle fracture, physiotherapy, rehabilitation, functional outcome

## Abstract

**Background:**

In current literature, the benefit of postoperative physiotherapy versus postoperative instructions by treating specialist only remains unclear. The aim of this review is to systematically assess existing literature regarding the functional outcome of postoperative physiotherapy compared to postoperative instructions by treating specialist only in the rehabilitation of patients with an ankle fracture. The secondary aim is to determine if there is a difference in ankle range of motion, strength, pain, complications, quality of life, and patient’s satisfaction between these 2 rehabilitation methods.

**Methods:**

For this review, the PubMed/MEDLINE, PEDro, Embase, Cochrane, and CINAHL databases were searched for studies that compared postoperative rehabilitation groups.

**Results:**

The electronic data search detected 20 579 articles. After exclusion, 5 studies with a total of 552 patients were included. Overall, no significant benefit in functional outcome of postoperative physiotherapy was seen compared to the instructions-only group. One study even found a significant benefit in favor of the instructions-only group. An exemption for beneficial effect of the use of physiotherapy could be made for younger patients, as 2 studies described younger age as a factor for better outcomes (functional outcome and ankle range of motion) in the postoperative physiotherapy group. Patients’ satisfaction, described by one study, was found to be significantly higher in the physiotherapy group (*P* = .047). All other secondary aims showed no significant difference.

**Conclusion:**

Because of the limited number of studies and the heterogeneity among studies, a valid conclusion about the general effect of physiotherapy cannot be formed. However, we identified limited evidence suggesting a possible benefit of physiotherapy in younger patients with an ankle fracture in functional outcome and ankle range of motion.

## Introduction

Ankle fractures are the most common fractures of the lower extremity, with approximately 169 per 100 000 persons per year sustaining this type of fracture. Ankle fractures are especially prevalent among young men and older women.^4,7,9^ After sustaining an ankle fracture, adults most often experience a rapid initial recovery, but additional functional improvement declines over time. On average, it is suggested that no further improvement can be expected after 24 months.^3^ Furthermore, functional outcome following treatment for an ankle fracture varies among fracture type, for example Weber C type fractures have a well-known worse outcome compared to Weber A and B type fractures.^30^

Treatment of patients with ankle fractures can either be conservative or operative, depending on the type of fracture and this is often followed by a period of immobilization.^11^ However, when treating ankle fractures, early rehabilitation is highly preferable.^17^ It is a general belief that rehabilitation reverses the decrease in muscle performance, functionality, and fatigue resistances induced by immobilization and the trauma itself.^27^ Postoperative rehabilitation can be performed in several ways, most often directed by a physiotherapist or postoperative instructions by treating specialist only.^20^

In current literature, the potential benefit of postoperative physiotherapy (PT) compared to postoperative instructions by a treating specialist only (ITS) in patients with an ankle fracture remains unclear. The primary aim of this systematic review is to systematically assess existing literature regarding the functional outcome of postoperative PT compared with postoperative ITS in the rehabilitation of patients with an ankle fracture. The secondary aim is to determine if there is a difference in ankle range of motion, strength, pain, complications, quality of life, and patients’ satisfaction between these 2 rehabilitation methods.

## Methods

### Search Strategy and Selection Criteria

This review is performed according to the Preferred Reporting Items for Systematic Reviews and Meta-Analyses (PRISMA) guidelines.^23^ Databases were searched for studies that compared postoperative rehabilitation by a physiotherapist and postoperative ITS in patients with an ankle fracture. PubMed/MEDLINE, PEDro, Embase, Cochrane, and CINAHL databases were searched up to October 2, 2022, to identify relevant RCTs and observational studies. Articles written in English, German, and Dutch were included and all patients included in these studies had to be 18 years or older. Abstracts for conferences, study protocols, letters, and comments were excluded. In addition, articles that focused on patients with a sprained ankle and patients with multiple injuries were also excluded. An information specialist at the Radboud University Medical Centre was consulted to assist in creating the search syntaxes (Appendix A). All retrieved articles were screened on title and abstract. Hereupon, potential suitable studies were read in full by 4 independent reviewers. Inclusion differences in articles were discussed and reference checking of included studies was applied.

### Quality Assessment

For included studies, the methodologic quality was independently assessed by 2 reviewers using the Methodological Index for Non-Randomized Studies (MINORS) (Appendix B). MINORS is a validated instrument designed to assess the methodologic quality and clear reporting of observational studies. MINORS is externally validated using randomized controlled trials, and therefore also appropriate to assess the quality of randomized controlled trials. This score ranges from 0 to 24, and the higher the score, the higher the quality.^28^

### Data Extraction

Study characteristics (author, year of publication, and study design) and patient demographics (fracture type and the duration of follow-up) were extracted. The number of included patients per group, age, sex, whether weightbearing or mobilization was allowed during rehabilitation, and duration of immobilization were extracted.

### Outcome Measures

The primary outcome is the patient’s functional outcome after surgical treatment to determine the effectiveness of the 2 rehabilitation strategies. This was evaluated by assessing various functional outcome scores as used by the included studies. Functional outcomes measured up to 6 months postoperative were defined as short term; after 6 months postoperation, it was defined as long term.

The secondary outcomes included in this review are ankle range of motion, strength, pain, complications, quality of life, and patient’s satisfaction.

## Results

### Data Search

**Figure 1.**
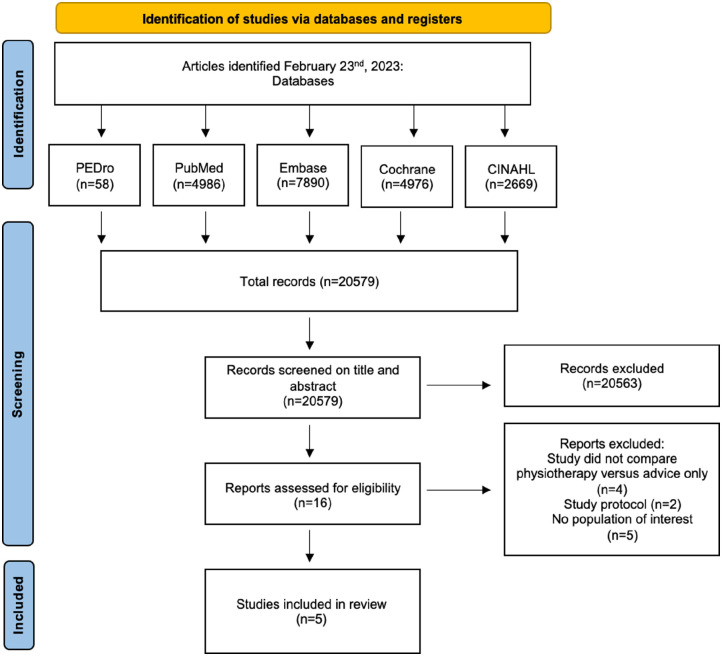
PRISMA flow diagram February 23, 2023.

A total of 20 579 articles were detected by electronic searches on February 23, 2023. After title and abstract screening, a total of 16 articles remained for full text eligibility assessment. Five exclusions were studies without the population of interest of this study, 2 studies were only a study protocol, and 4 studies had a noncomparative study design (Figure 1). Reference checking did not result in additional suitable studies for inclusion. Five studies were considered suitable and subsequently included in this systematic review.^6,10,16,24,25^

### Quality Assessment

The mean MINORS score (±SD) is 18.8 (±2.28). Karam et al^16^ had the lowest score of 15, and Nilsson et al^25^ had the highest score of 21. Appendix C shows the distribution of the quality of the studies.

### Study and Patient Characteristics

Two randomized controlled trials, 2 prospective observational studies, and 1 retrospective study were included (Table 1). Table 1 summarizes these 5 studies. In total, 552 patients were included. A total of 264 patients received PT and 288 patients received ITS. Inclusion rates varied from 45 to 214 patients included per study. No statistically significant differences were seen among the main characteristics of the groups in the included studies. The mean age of the included patients ranged from 39 to 53 years. Karam et al^16^ included only patients with a Weber B ankle fracture. Other studies included patients with various types of ankle fractures (uni-, bi-, and/or trimalleolar) (Table 1).^6,10,25^ Four studies included operatively treated patients only.^6,10,16,25^ Moseley et al^24^ included both operated and nonoperatively treated patients. Büker et al^6^ studied rehabilitation directly after surgery, whereas all other studies^10,16,24,25^ incorporated a period of immobilization. When applied, the average duration of immobilization was 6 weeks. The duration of the follow-up period ranged from 6 to 36 months.

**Table 1.** Included Studies and Baseline Characteristics.

Study	Year	Study Design	Ankle Fracture Type	Follow-up, mo	Groups	Postoperative Policy	Duration of Immobilization, d (±SD)	Number of Patients	Age, y (mean ±SD)	Male/ Female
Büker et al^6^	2019	Prospective observational study	Medial, lateral and bimalleolar	28	Physiotherapy	Mobilization and weightbearing	0	35	39.23 (11.72)	24/11
					Instructions only			73	41.78 (13.70)	51/22
Ferguson et al^10^	2019	Prospective observational study	Bimalleolar or trimalleolar with or without dislocation	6	Physiotherapy	NR	NR	38	52.6 (11.7)	8/30
					Instructions only			42	50.9 (17.1)	15/27
Karam et al^16^	2017	Cross-sectional retrospective population study	Isolated unimalleolar Weber B	36	Physiotherapy	NR	42	35	NR	NR
					Instructions only			10	NR	NR
Moseley et al^24^	2015	RCT	Isolated and uncomplicated	6	Physiotherapy	Mobilization and weightbearing	45.3 (11.2)	106	43.1 (16.5)	43/63
					Instructions only	mobilization	47.9 (13.2)	108	41.3 (15.2)	51/57
Nilsson et al^25^	2009	RCT	Ankle fractures (all types)	12	Physiotherapy	Mobilization and weightbearing	43 (5.5)	50	45 (22)	19/31
					Instructions only			55	43 (22)	24/31
					Instructions only			73	41.78 (13.70)	51/22

Abbreviations: NR, not reported; RCT, randomized controlled trial.

### Description of the Intervention

The frequencies of the physiotherapy sessions varied from one to 3 times a week and complete duration of the therapy ranged from 4 to 12 weeks.^6,24,25^ Standardized physiotherapy protocols were used in 2 studies, namely Nilsson et al^25^ and Moseley et al.^24^ Nilsson et al described more exercises and a total of 8 more weeks than Moseley et al. Only Nilsson et al described the main goals for the rehabilitation process as well as main goals for the exact physical outcomes. Büker et al^6^ used the same exercise program for both groups, either under supervision of a physiotherapist or at home without supervision. Karam et al^16^ and Ferguson et al^10^ did not describe the exact rehabilitation protocol.

### Functional Outcomes

#### American Orthopaedic Foot & Ankle Society score

Both Karam et al^16^ and Büker et al^6^ used the American Orthopaedic Foot & Ankle Society (AOFAS) score.^32^ Büker et al^6^ did not report the specific timing of the AOFAS score measurement but described the mean functional outcomes over a certain follow-up period. In this study, the average follow-up period was 27.9 ± 9.9 months. There was a significantly higher AOFAS score in the ITS group compared to the PT group (83.8 ± 15.2 vs 76.6 ± 17.5) (Appendix D).

Karam et al^16^ showed no significant difference in the mean functional outcome using the AOFAS at the 6-month and 3- year follow-up between the 2 rehabilitation groups.^6^

#### Olerud Molander Ankle Score

Karam et al. also used the Olerud Molander Ankle Score (OMAS).^22^ Mean scores were measured at 6 months, 1 year, 2 years, and 3 years. There was no significant difference between the PT and the ITS group.^16^

Nilsson et al^25^ showed only significant difference when the groups were subdivided by age. The PT group demonstrated significantly higher scores compared to the ITS group in subjects younger than 40 years (86.5 ± 12.4 vs 72.8 ± 17.6 at 12 months and 78.1 ± 15.7 vs 65.5 ± 15.4 at 6 months).^25^

#### LEFS, FAAM, and SFMA

Moseley et al^24^ was the only one using the Lower Extremity Functional Scale (LEFS).^5^ Analysis showed no significant benefit of PT over the ITS group.^24^ Ferguson et al^10^ used the Foot and Ankle Ability Measure (FAAM) scale and the Selective Functional Movement Assessment (SMFA) score.^21,26^ In both self-reported outcome instruments, there was no significant difference between the PT group and the ITS group.^10^

### Ankle Range of Motion

In the included studies, the range of motion was the most common outcome measure, apart from the primary outcome measures.^6,24,25^ Nilsson et al^25^ measured an angle in degrees to express the range of motion; the normal value of dorsiflexion was stated as 30 degrees. A significant difference was seen, when divided by age group, in favor of the PT group in subjects younger than 40 years in the plantar flexors (at 6 months 45 ± 5.9 degrees vs 43 ± 7.0 degrees), at 12 months 48 ± 5.9 degrees vs 47 ± 6.0 degrees) and dorsal flexors (at 6 months 30 ± 5.7 degrees vs 30 ± 6.5 degrees, at 12 months 33 ± 5.7 degrees vs 32 ±v 6.6 degrees) (Table 2).^25^ Büker et al^6^ stated that plantar flexion in the PT group was better than plantar flexion in the ITS group in patients younger than 40 years (*P* = .045) (Table 2).

**Table 2.** Secondary Outcome Measure Ankle Range of Motion.

	Ankle Range of Motion (<6 mo), Degrees, Mean ± SD				Ankle Range of Motion (>6 mo), Degrees, Mean ± SD			
Study	Plantarflexion	Dorsiflexion	Eversion	Inversion	Plantarflexion	Dorsiflexion	Eversion	Inversion
Büker et al,^6^ 2019								
Physiotherapy	NR	NR	NR	NR	33.24 ± 9.09	11.29 ± 8.89	12.44 ± 9.36	19.29 ± 10.47
Instructions only	NR	NR	NR	NR	33.24 ± 8.02	14.40 ± 7.80	12.12 ± 7.39	17.52 ± 8.73
Moseley et al,^24^ 2015								
Physiotherapy	NR	37.4 (47.6) (mm)	NR	NR	NR	NR	NR	NR
Instructions only	NR	30.4 (51.3) (mm)	NR	NR	NR	NR	NR	NR
Nilsson et al,^25^ 2009								
Physiotherapy	44 ± 6.3	28 ± 6.5	NR	NR	47 ± 6.1	30 ± 6.1	NR	NR
Instructions only	42 ± 6.6	27 ± 6.6	NR	NR	45 ± 5.7	29 ± 7.1	NR	NR

Abbreviation: NR, not reported.

### Pain

Moseley et al^24^ and Büker et al^6^ both described pain as an outcome measure. Both studies measured pain using a numeric rating scale ranging from 0 to 10, a higher score meaning more pain. Although various aspects of pain were analyzed (eg, pain during (im)mobilization, impact on daily life), no statistically significant difference for pain between the PT group and the ITS group was found.^6,24^

### Complications

Two studies described postoperative complications for both rehabilitation methods. Nilsson et al^25^ described 4 complications in the PT group, consisting of superficial infections or deep vein thrombosis compared to 1 complication, a superficial infection, in the ITS group. Fergusson et al^10^ looked at 7 different kinds of complications. Both studies^10,25^ did not find a significant difference in the number of complications between the 2 rehabilitation groups.

### Patients’ Satisfaction and Quality of Life

Moseley et al^24^ evaluated patient satisfaction on a 5-point Likert scale ranging from 1 (extremely dissatisfied) to 5 (extremely satisfied). Participants in both groups were generally satisfied with the intervention they received. There was no significant difference between the 2 rehabilitation groups. Büker et al^6^ described surgical satisfaction and rehabilitation satisfaction. It was evaluated with the use of a numeric scale (0-10). Rehabilitation satisfaction of the PT group was statistically significantly higher (*P* = .047) compared to the ITS group at the average follow-up period of 28 months, but there was no significant difference when adjusting for age.^6^

Moseley et al^24^ was the only one using the Assessment of Quality of Life.^13^ At all follow-up moments, none of these differences were significant. Nilsson et al^25^ and Büker et al^6^ used the 36-Item Short Form Health Survey (SF-36). Nilsson et al^25^ only analyzed physical health and mental health domains of the SF-36. Both physical health and mental health showed no significant difference between the 2 groups.

## Discussion

After systematically reviewing all articles concerning postoperative rehabilitation of patients sustaining an ankle fracture, this study found no clear benefit of PT compared with ITS in terms of functional outcome, ankle range of motion, pain, complications, and patient outcomes. We identified limited evidence suggesting a possible benefit of physiotherapy in younger patients with an ankle fracture in functional outcome and ankle range of motion.

Remarkably, Büker et al^6^ was the only published study that showed better results for the ITS group. Among other reasons, this might be caused by postoperative immobilization because Büker et al was the only study that included no cast immobilization after surgery. A recent study^29^ showed that postoperative unprotected weightbearing and mobilization improves short-term functional outcome. However, the effect of this on the comparison between PT and ITS remains unclear. It is suggestable that patients without cast immobilization experience less stiffness in the ankle joint compared to the patients with cast immobilization. As a consequence, patients in the ITS group could start immediately with their rehabilitation, whereas the PT group must wait for the physiotherapeutic sessions.

The fact that this review found no benefit of PT over ITS could be caused by the exact type of treatment that is provided by the physiotherapist. This review showed a substantial heterogeneity among the various physiotherapy treatment regimens. Moreover, it is not clear if this treatment is provided in line with the current evidence-based rehabilitation protocols. In addition, Zadro et al,^33^ which described the percentage of physiotherapists who provide recommended evidence-based treatments to patients, concluded that a lot of physiotherapists appear not to follow evidence-based guidelines when treating musculoskeletal conditions. Zadro et al stated that the contribution of physiotherapy could be increased by providing the treatments as they are recommended in the guidelines.

Out-of-trial physiotherapy utilization could be another explanation for the lack of benefit for patients who received postoperative physiotherapy. Up to 76% out-of-trial physiotherapy was described by the 2 largest studies in this review. This could lead to a substantial underestimation of the effect of physiotherapy.^24,25^ Patients seeking professional rehabilitation assistance, although not prescribed by their treating physician, might come from a general assumption among patients that physiotherapy is beneficial. A study performed by van Harten et al^31^ showed that 89% of all patients with an ankle fracture believed that physiotherapy is necessary for pain relief and improved function.

Büker et al^6^ showed better patient satisfaction in the physiotherapy group, and it suggested that this is possibly due to socioemotional factors between the patient and therapist (ie, positive social connection, communication, empathy, and mutual respect). This might be an important direction for future research, because there is a growing amount of evidence suggesting a relation between psychological factors and clinical outcome after trauma.^1,14,19^ For instance, patients with higher anxiety levels might benefit more from physiotherapy to overcome their kinesiophobia and regain mobility.^8^ In line with this is a review that studied the role of therapeutic alliance in musculoskeletal physiotherapy. Therapeutic alliance is the working relationship or positive social connection between a patient and a therapist.^15^ It is suggested that enhanced therapeutic alliance has a beneficial effect on treatment adherence, pain, and physical functioning.^2,12^ Among other reasons, this therapeutic alliance could partially explain the age-related differences in this review. A study showed that age moderated the relationship between therapeutic alliance and clinic-based adherence, where younger and more autonomous individuals are more adherent to therapy.^18^

In this review and the included studies, several limitations were identified. First, significant differences between PT and ITS were seen, whereas absolute differences were often marginal. Therefore, clinical relevance remains questioned. Second, it was impossible to take specific patient or fracture characteristics into account. For instance, in case of type of fracture, it could be suggested that specific types of fractures could potentially benefit more from postoperative physiotherapy. Also, patients’ needs could not be taken into account. Presumably professional athletes or workers in heavy labor could regain more functionality with physiotherapy. Further, Moseley et al^24^ included patients with and without surgical fixation; it was included in this systematic review based on the substantial amount of operatively treated ankle fractures as well as the limited amount of studies available on postoperative rehabilitation. It is not described in the article if there was a difference in outcome between these groups.

A strength of this systematic review is that because of the broad search string in different databases and the inclusion of all available relevant study designs, the risk of publication bias is decreased to a minimum. This is also the first systematic review specifically about this subject. There were also no competing interests that could have led to bias in favor of a particular intervention.

In summary, because of the limited number of studies and the heterogeneity, no conclusion can be made up about the effect of physiotherapy. However, some evidence shows that selective groups of patients might benefit from physiotherapy as well as instructions by the treating specialist only, which mandates a more patient-tailored approach. To date, there is only limited evidence concerning the influence of patient, fracture, and/or treatment characteristics on functional outcomes after operatively managed ankle fractures, and the role of subsequent physiotherapy. This systematic review of a limited number of studies revealed a possible functional benefit for young patients with ankle fractures treated with physiotherapy.

## Appendix A: Search Syntaxes

**PEDro** (n = 57)

Title/abstract: Ankle fracture

Body part: ankle/foot

**PubMed** (n = 4857)

(“ankle fractures”[MeSH Terms] OR “fibula/surgery”[MeSH Terms] OR “tibia/surgery”[MeSH Terms] OR ((“ankle*”[Title/Abstract] OR “fibula*”[Title/Abstract] OR “tibia*”[Title/Abstract] OR “malleol*”[Title/Abstract] OR “trimalleolar”[Title/Abstract] OR “bimalleolar”[Title/Abstract]) AND (“fracture*”[Title/Abstract] OR “surg*”[Title/Abstract]))) AND (“rehabilitation”[MeSH Terms] OR “rehabilitation”[MeSH Subheading] OR “physical therapy modalities”[MeSH Terms] OR “physical therapy specialty”[MeSH Terms] OR “directed exercis*”[Title/Abstract] OR “exercise therap*”[Title/Abstract] OR “manipulation therap*”[Title/Abstract] OR “manipulative therap*”[Title/Abstract] OR “musculoskeletal manipulation*”[Title/Abstract] OR “physical therap*”[Title/Abstract] OR “physiotherap*”[Title/Abstract] OR “rehabilitation”[Title/Abstract] OR “remedial exercis*”[Title/Abstract] OR “supervised exercis*”[Title/Abstract] OR “supervised training*”[Title/Abstract])

**EMBASE** (n = 7556)

(ankle fracture/ OR exp malleolus fracture/ OR exp fibula fracture/ OR exp fibula/su OR tibia fracture/ OR exp distal tibia fracture/ OR tibia/su OR exp distal tibia/su OR tibial epiphysis/su OR tibial metaphysis/su OR ((ankle* OR fibula* or tibia* OR malleol* OR trimalleolar OR bimalleolar) AND (fracture* OR surg*)).ti,ab,kw.) AND (exp rehabilitation/ OR exp physiotherapy/ OR exp exercise/ OR rehabilitation.fs. OR (directed exercis* OR exercise therap* OR manipulation therap* OR manipulative therap* OR musculoskeletal manipulation* OR physical therap* OR physiotherap* OR rehabilitation OR remedial exercis* OR supervised exercis* OR supervised training*).ti,ab,kw.) Filter: no conference abstracts

**Cochrane** (n = 4781)

((ankle* OR fibula OR tibia* OR malleol* OR trimalleolar OR bimalleolar):ti,ab,kw) AND ((directed exercis* OR exercise therap* OR manipulation therap* OR manipulative therap* OR musculoskeletal manipulation* OR physical therap* OR physiotherap* OR rehabilitation OR remedial exercis* OR supervised exercis* OR supervised training*):ti,ab,kw)

**CINAHL** (n = 2610)

((MH “ankle fractures”) OR (MH “fibula fractures”) OR (MH “tibial fractures+”) OR (MH “Fibula/SU”) OR (MH “tibia/SU”) OR TI ((ankle* OR fibula OR tibia* OR malleol* OR trimalleolar OR bimalleolar) AND (fracture* OR surg*)) OR AB ((ankle* OR fibula OR tibia* OR malleol* OR trimalleolar OR bimalleolar) AND (fracture* OR surg*)) ) AND ((MH “rehabilitation+”) OR (MW RH) OR (MH “exercise+”) OR TI (”directed exercis*” OR “exercise therap*” OR “manipulation therap*” OR “manipulative therap*” OR “musculoskeletal manipulation*” OR “physical therap*” OR “physiotherap*” OR “rehabilitation” OR “remedial exercis*” OR “supervised exercis*” OR “supervised training*”) OR AB (”directed exercis*” OR “exercise therap*” OR “manipulation therap*” OR “manipulative therap*” OR “musculoskeletal manipulation*” OR “physical therap*” OR “physiotherap*” OR “rehabilitation” OR “remedial exercis*” OR “supervised exercis*” OR “supervised training*”))

## Appendix B

**Table:** MINORS Criteria.

Methodological items	2	1	0
A clearly stated aim	Aim or hypothesis including outcomes have been reported	Aim or hypothesis have been reported without a clear outcome	Not reported
Inclusion of consecutive patients	Explicit inclusion and exclusion criteria have been reported	Unclear or poor description inclusion and exclusion criteria have been reported	Not reported
Prospective collection of data	Prospective	Retrospective	Not reported
Endpoints appropriated to the aim of the study	Outcomes are appropriate to the aim of the study	Outcomes are not appropriate to the aim of the study	Not reported
Unbiased assessment of the study endpoint	Blind evaluation of objective outcomes and double-blind evaluation of subjective outcomes	One or more outcomes have been blinded	Blinding has not been performed or is not reported
Follow-up period appropriate to the aim of the study	≥1 y	<1 y	Not reported
Loss to follow-up	≤5%	>5% and ≤20%	Not reported or more than 20%
Prospective calculation of the study size	Power analysis has been performed	Explanation for the number of included patients without a power analysis	Not reported or not performed
An adequate control group	Physiotherapy compared with instructions by treating specialist or physiotherapist only	Not applicable	Not reported
Contemporary group	Study group and controls have been managed during the same time period	Study groups and controls have not been managed during the same time period	Not reported or unclear description
Baseline equivalence of groups	Baseline characteristics have been described for both groups and are comparable	Baseline characteristics have not been described thoroughly or are not comparable	Not reported
Adequate statistical analyses	Statistical analysis has been described including type of test	Inadequate statistical analysis	Not reported

## Appendix C

**Table:** Total Quality MINORS Score per Study.

Criteria	Büker et al^6^	Ferguson et al^10^	Karam et al^16^	Moseley et al^24^	Nilsson et al^25^
A clearly stated aim	2	2	2	2	2
Inclusion of consecutive patients	2	2	1	2	2
Prospective collection of data	2	2	1	2	2
Endpoints appropriated to the aim of the study	2	2	2	2	2
Unbiased assessment of the study endpoint	1	0	1	0	1
Follow-up period appropriate to the aim of the study	2	1	2	1	2
Loss to follow-up	1	2	0	0	1
Prospective calculation of the study size	0	0	0	2	2
An adequate control group	2	2	1	2	1
Contemporary group	2	2	2	2	2
Baseline equivalence of groups	2	2	1	2	2
Adequate statistical analyses	2	2	2	2	2
Total quality score MINORS	20	19	15	19	21

## Appendix D

**Table:** Functional outcome measures.

Study	Functional Scores, Short-term (<6 mo)		Functional Scores, Long-term (>6 months)	
	Physiotherapy	Instructions Only	Physiotherapy	Instructions Only
Büker et al,^6^ 2019	NR	NR	AOFAS 76.63 (±17.46 SD)	AOFAS 83.75 (±15.15 SD)
Ferguson et al,^10^ 2019	FAAM 69.7 SMFA 20.1	FAAM 70.9 SMFA 24.4	NR	NR
Karam et al,^16^ 2017	NR	NR	AOFAS 80 OMAS 79 VAS-FA 73	AOFAS 78 OMAS 75 VAS-FA 85
Moseley et al,^24^ 2015	LEFS 69.9 (95% CI 67.4-72.5)	LEFS 69.9 (95% CI 67.3-72.5)	NR	NR
Nilsson et al,^25^ 2009	OMAS 62.4 (SD 25.1)	OMAS 63.5 (SD 20.9)	OMAS 74.4 (SD 19.7)	OMAS 71.4 (SD 22.3)

Abbreviation: NR, Not reported.
